# Four Million Pigs with Bad Grammar and Worse Logic, but No Disease

**Published:** 1884-03

**Authors:** 


### Four Million Pigs with Bad Grammar and Worse Logic, But No Disease.

Under the heading of "American Retaliation," the *British Medical Journal*, of February 9, 1884, prints the following paragraph:

"Although it has been proved, beyond the possibility of doubt, that ordinary cooking is a certain protection against the ill effects of trichinous pork; and that with a few quite insignificant exceptions, those people only have suffered who, like the Germans and Swedes, are in the habit of consuming bacon and ham in the raw state; and moreover, that, while the disease is very general among the Russian and German animals, no single case has been shown to have been caused by the use of American meat, several of the continental states, as France, Germany, Italy, and Austria, have been induced, by the agitation set up by interested traders, on the pretext of regard for the public health, to prohibit importation from the United States. Our transatlantic cousins have hit on a means of revenging themselves; and have introduced into Congress a bill to stop the importation of wines, liqueurs, etc., from all countries imposing restrictions on their trade in hams, etc., so long as these restrictions remain in force. At the same time the authorities at Chicago have determined on establishing a body of inspectors; but it is very doubtful whether much real security can be thus given, since not fewer than four million pigs are slaughtered there annually, at the rate of 1,400 per hour."

These words read to us as though they had been penned in Dublin rather than in London. If "no single case (of trichinosis) has been shown to have been caused (sic) by the use of American meat:" and if the disease is "very general among the Russian and German animals," why should the efforts made by the Chicago authorities to protect those who refuse to protect themselves by cooking the flesh of the swine before it is eaten be regarded as doubtful of effect?

Retaliation, moreover, is not revenge. The purpose of those American legislators who favor the passage of laws unfavorable to the importation into this country of wines and liqueurs, is not to punish, but to persuade; not to revenge nor to retaliate, but to teach the lessons of reciprocity.

